# Biomineralization of Collagen-Based Materials for Hard Tissue Repair

**DOI:** 10.3390/ijms22020944

**Published:** 2021-01-19

**Authors:** Le Yu, Mei Wei

**Affiliations:** 1 Department of Chemical and Biomolecular Engineering, Ohio University, Athens, OH 45701, USA; yul@ohio.edu; 2 Department of Mechanical Engineering, Ohio University, Athens, OH 45701, USA

**Keywords:** biomineralization, intrafibrillar, extrafibrillar, collagen, tissue engineering

## Abstract

Hydroxyapatite (HA) reinforced collagen fibrils serve as the basic building blocks of natural bone and dentin. Mineralization of collagen fibrils play an essential role in ensuring the structural and mechanical functionalities of hard tissues such as bone and dentin. Biomineralization of collagen can be divided into intrafibrillar and extrafibrillar mineralization in terms of HA distribution relative to collagen fibrils. Intrafibrillar mineralization is termed when HA minerals are incorporated within the gap zone of collagen fibrils, while extrafibrillar mineralization refers to the minerals that are formed on the surface of collagen fibrils. However, the mechanisms resulting in these two types of mineralization still remain debatable. In this review, the evolution of both classical and non-classical biomineralization theories is summarized. Different intrafibrillar mineralization mechanisms, including polymer induced liquid precursor (PILP), capillary action, electrostatic attraction, size exclusion, Gibbs-Donnan equilibrium, and interfacial energy guided theories, are discussed. Exemplary strategies to induce biomimetic intrafibrillar mineralization using non-collagenous proteins (NCPs), polymer analogs, small molecules, and fluidic shear stress are discussed, and recent applications of mineralized collagen fibers for bone regeneration and dentin repair are included. Finally, conclusions are drawn on these proposed mechanisms, and the future trend of collagen-based materials for bone regeneration and tooth repair is speculated.

## 1. Introduction

Natural bone is an organic and inorganic composite constituting of mineralized collagen fibrils, which serves as the second level of its seven hierarchical levels. The inner dentin-pulp layer of human tooth has a similar mineralization profile to native bone [1]. The main inorganic component in these tissues is nanosized hydroxyapatite (HA; Ca_10_(PO_4_)_6_(OH)_2_). Its crystal structure ascribes to the hexagonal crystal system, which enables it with structural and compositional flexibility and stability. It allows for an abundant variety of ion substitutions involving divalent or trivalent cations for the Ca^2+^ sites while anions for the phosphate and hydroxyl sites. For examples, carbonate-substituted (carbonated) hydroxyapatite forms the basic mineral component of hard tissues in vertebrates [2]; fluoride-substituted hydroxyapatite (fluorapatite) is found in the teeth of sharks and other fishes [3]. It is a common strategy to use transition metals, such as Mn, Fe, Co and Ni, to substitute Ca(I) (surrounded by phosphate), and Ca(II) (surrounded by hydroxyl) in the HA lattice to grant materials with magnetic properties to be used in many biomedical applications, such as drug delivery, diagnostic testing, and magnetic resonance imaging [4,5,6].

The main organic component in bone and dentin is type I collagen, which has a complex structure including four levels of hierarchy. The primary level is amino acid triplets consisting mostly of proline (Pro), hydroxyproline (Hyp), and glycyl (Gly), following the pattern of Gly-Pro-X or Gly-X-Hyp, where X can be any of other amino acid species [7]. The amino acid triplets stack repeatedly to form its secondary level of structure [8]. The tertiary level is the well-known triple helix consisting of three interconnected α-chains [9]. The last hierarchy is in supermolecular level that forms collagen fibrils or fibers [10]. These collagen fibrils then continue to self-assemble both linearly and laterally into a hydrogel network under suitable physicochemical conditions [11]. As a naturally extracted polymer from extracellular matrix (ECM), collagen-based materials demonstrate excellent biocompatibility, which supports cellular adhesion, proliferation, migration and differentiation. As a result, they have been widely used in many biomedical applications, such as drug delivery, tissue engineering, wound healing, and cosmetic surgery [8,12,13].

Bone tissue engineering aims to repair or regenerate native bones in defective areas that are beyond their self-healing capabilities with the aid of grafting materials, typically scaffolds, progenitor cells and/or growth factors. Thus, selection of scaffolds becomes the key to the success of bone tissue engineering [14]. First, the material should be biocompatible and not induce any adverse effects or toxicity to the host. Second, it should be biodegradable to avoid secondary surgery after healing. Third, it should have interconnected pores to support cell adhesion, vascularization and transportation of nutrients and metabolic wastes. Finally, it should possess suitable mechanical properties to against various stresses and maintain structural integrity [8,15]. Collagen-hydroxyapatite-based (Col-HA-based) biomimetic scaffolds that structurally and compositionally resemble the natural bone have drawn extensive attention from researchers because of their excellent biocompatibility, biodegradability, low antigenicity, and compositional and structural flexibility. In contrast to bone, dental hard tissues, such as the outmost enamel and inner dentin-pulp complex, cannot self-heal if damaged. The cause of dental caries is a dynamic process of imbalance between demineralization and remineralization [16]. The caries is typically filled with artificial materials, such as amalgam, ceramics and resin composites. However, these materials could also bring about side effects, including trauma to sound dental tissue, dentin hypersensitivity, and interface microleakage between the filling materials and dental tissue [17]. Thus, biomimetic Col-HA-based materials provide a promising alternative for accelerating remineralization of dentin for tooth defect repair at an early stage of pathological conditions [18]. Overall, biomimetic mineralization of collagen becomes an essential strategy for developing bioinspired materials for both orthopedic and dental applications.

Existing reviews about collagen-based materials have been focused on (1) the collagen sources, such as bovine tendons, rat tails, and fish, (2) the physical forms and morphologies, such as hydrogels, fibers, microspheres, and scaffolds or (3) their applications, such as tissue engineering, wound healing, and cosmetic surgery. Thus, there is a need to develop a comprehensive review on these mechanisms, especially to include the most recent progress of biomineralization mechanisms. In this review, both intrafibrillar and extrafibrillar mineralization mechanisms are discussed and strategies for biomimetic mineralization of collagen-based materials are reviewed, and their applications in bone tissue engineering and tooth remineralization are covered. The future trend of mineralized collagen-based material in tissue engineering and dentin repair is speculated.

## 2. Biomineralization of Collagen

**Figure 1:**
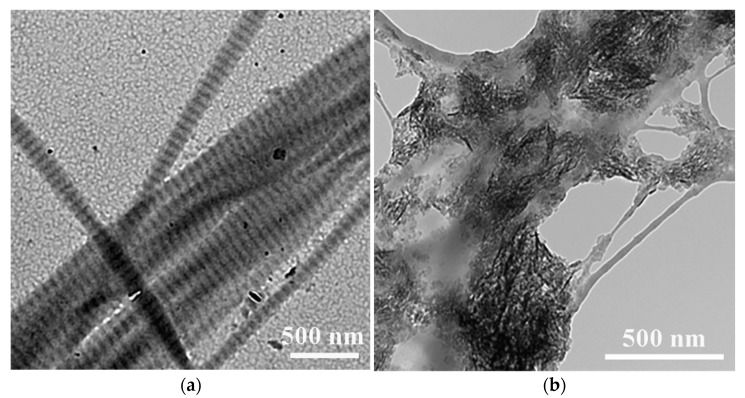
Typical transmission electron microscopy (TEM) images of (**a**) highly oriented intrafibrillar (showing clear D-banding) and (**b**) extrafibrillar mineralized collagen fibrils. Images are modified from [24] with permission. Copyright @ John Wiley & Sons, Inc., Hoboken, NJ, USA.

Ordered biomineralization holds the basis of structural organization and functionality for multiple organisms. In comparison, pathological mineralization leads to the formation of undesired biominerals, such as kidney stones and dental calculus [19]. It has been recently reported that deficiency of pyrophosphate, a known endogenous biomineralization inhibitor, could result in abnormal calcification process such as ectopic vascular calcification in the form of hydroxyapatite crystals and thus increase the risk of cardiovascular disease [20,21,22]. Developing an in-depth understanding of biomineralization mechanism(s) is therefore imperative for promoting the desired mineralization while minimizing the undesired ones. It will also shed light on synthetic chemistry to develop bioinspired materials via biomimetic mineralization. As shown in Figure 1, the biomineralization of type I collagen, in terms of the distribution patterns of hydroxyapatite in collagen, can be distinguished into intrafibrillar mineralization, where HA exhibits within the gap zone of collagen fibrils, and extrafibrillar mineralization when HA deposits on the surface of collagen fibrils. However, the mechanisms driving these processes remain unclear and debatable [19,23].

**Figure 2:**
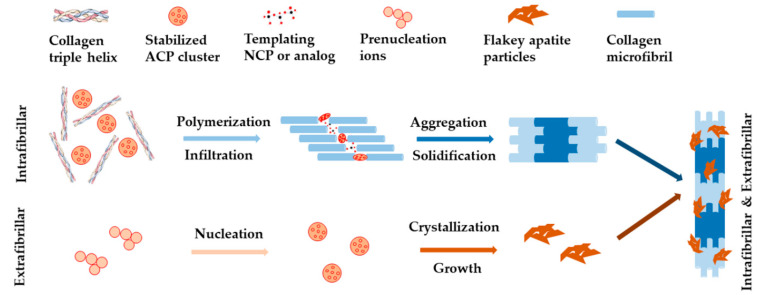
Schematic illustration of non-collagenous proteins (NCPs)-regulated collagen biomineralization with both intrafibrillar and extrafibrillar mineralization according to the non-classical theory.

The classical view of biomineralization relies on crystallization pathway, which describes the movement of ions from their original source into targeted tissue forming mineralized bone and dentin. In this process, cells (or bacteria) are believed to play essential roles, by temporarily concentrating inorganic ions in intracellular membrane-bound vesicles in the form of a highly disordered solid phase. The solid phase is then destabilized and crystallized into minerals when it is transported into the final mineralization position [25]. Challenging this, a newer standpoint of biomineralization came from the non-classical theory, which was built upon the discovery of prenucleation clusters in the process of calcium carbonate-based biomineralization [26]. For example, it was proposed that the regulation of calcium phosphate (CaP) phases exhibited two major processes, namely the sequestration of calcium and phosphorous ions into fluidic amorphous calcium phosphate (ACP) precursors, and the templating of these clusters into ordered forms [27,28]. Both processes were believed to be well regulated by extracellular non-collagenous proteins (NCPs) and other factors such as biomolecules [29,30]. As shown in Figure 2, with these regulations, HA nanocrystals deposited within the gap zone of collagen fibrils, forming intrafibrillar mineralization, at an early stage of the biomineralization process. Then they precipitated on the surface of collagen fibrils, forming extrafibrillar mineralization, at a later stage of biomineralization. These processes led to collagen fibrils with both intrafibrillar and extrafibrillar mineralization [24,31,32]. The mineralized fibrils further bundled into highly ordered and stacked structure, forming either compact or spongy bone [33].

The role that collagen plays during biomineralization process has also been advanced with the development of better understanding of the system. Collagen was long believed to only serve as a structural matrix and considered to be inactive in biomineralization. At the end of last decade, collagen was discovered to play an active role in the apatite mineralization process. This was supported by the discovery of a positively charged region existing in collagen fibrils at the interface of the gap and overlap zones [19,31]. By systematically analyzing the primary amino acid sequence of human type I collagen, it was revealed that collagen is a biomacromolecule enriched with multiple charged amino acids [34]. Therefore, the existence of positively charged domains in collagen fibrils provides binding sites for templating NCPs, which further contributes to the regulation of mineral deposition in an ordered manner. In a recent endeavor of explaining the role of collagen molecules in mineral nucleation, a simulation study using all-atom Hamiltonian replica exchange molecular dynamics has been conducted [35] The study showed that the charged amino acid side chains in collagen molecules are oriented toward the fibril gap zones and played a significant role in templating the nucleation of ACP phase. Thus, at the atomic level, calcium phosphate was verified to nucleate primarily in these regions. Despite substantial merits and significance of these findings and proposed theories, most of these studies were conducted using simulations or simplified in vitro models, where more complicated in vivo biological environment was not taken into consideration.

## 3. Intrafibrillar Mineralization Mechanisms

In general, extrafibrillar mineralization of collagen takes place in the form of mineral deposition on the surface of collagen fibrils in the absence of nucleation inhibitors [36]. Based on the classical theory, apatite nucleates at suitable physicochemical conditions according to the following reactions [37]:

(1) $${\mathrm{HCO}}_{3}^{-}\;\to {\;\mathrm{CO}}_{2}+{\mathrm{OH}}^{-}$$

(2) $$5{\mathrm{Ca}}^{2+}+\;3{\mathrm{HPO}}_{4}^{2-}+\;4{\mathrm{OH}}^{-}\;\to {\;\mathrm{Ca}}_{5}{\left({\mathrm{PO}}_{4}\right)}_{3}\left(\mathrm{OH}\right)+\;3H_{2}O$$

when the pH of solution is brought up to an appropriate range with the decomposition of bicarbonate, HA starts to nucleate, and particles slowly deposit on the surface of collagen [38]. While based on the non-classical theory, prenucleation clusters and spherical ACP are formed as intermediate products before apatite crystals deposit and grow on collagen fibrils to form extrafibrillar mineralization [39]. Thus, the extrafibrillar mineralization of collagen is a relatively clear process and has been well studied [40,41]. In recent years, extensive studies have been focused particularly on the exploration of the mechanisms of intrafibrillar mineralization of collagen, which were mostly built upon the classical and the non-classical theory.

### 3.1. Capillary Action

Polymer induced liquid precursor (PILP) is considered to be one of the most important transient states before mineral infiltration into collagen fibrils and crystallization during intrafibrillar mineralization. The term “PILP” was first introduced by Gower et al. [42,43] along with the discovery of liquid-liquid phase separation of amorphous calcium carbonate (ACC) in the presence of polyanionic polyaspartic acid (PASP) under optical microscopy. They proposed that during the PILP process, the charged polymer acted as a processing-directing agent, with the aid of which the conventional solution crystallization process was converted into an amorphous liquid precursor-like process [44]. Using the formation of calcium carbonate and CaP based on PILP as examples, the dynamic compositional evolution and interactions between the polymer and the amorphous precursor have been revealed recently with the aid of advanced analytical techniques, such as cryogenic transmission electron microscopy (cryo-TEM), in situ atomic force microscopy (AFM), nuclear magnetic resonance (NMR), and modeling simulations [45,46,47,48]. A comprehensive understanding of PILP theory along with its applications for bone regeneration has been reported recently [49]. The basis for the PILP theory is the liquid-liquid phase separation as liquid or liquid-like component can take flexible shape prior to solidification, which largely facilitates the infiltration of minerals into collagen fibrils to form intrafibrillar mineralization.

Upon the proposal of PILP, Gower et al. [50,51] opined subsequently that a liquid phase mineral precursor could be drawn into the gap spaces of collagen fibrils by capillary action in the PILP process before it was solidified and crystallized into minerals, forming intrafibrillar mineralized collagen fibrils that were embedded with nano crystallites. The assumption was initially made based on their observations from capillary forces acting on the phase boundaries between the liquid precursor phase and collagen fibrils in a calcium carbonate system [50,52]. With the evolution of techniques and knowledge, they subsequently confirmed their theory by duplicating similar observations via a combination analyses of transmission electron microscopy (TEM), scanning electron microscopy (SEM), confocal microscopy, and X-ray diffraction (XRD) in a CaP system that is more closely mimicking the nanostructure of natural bone [29,44,53]. Briefly, they found that PASP (processing-directing agent) incorporated with calcium ions could only penetrate into turkey tendon for around 100 μm in comparison to PASP incorporated with CaP fluidic precursor that had a penetration depth over 500 μm. With a smaller Stokes radius, PASP + Ca would be expected to penetrate deeper into the dense-packed collagen scaffolds of turkey tendon than PASP + CaP if they migrated through ion diffusion [29]. Therefore, they suggested that a capillary action might have been involved in intrafibrillar mineralization as capillary forces are expected to provide rapid and long-range transportation of a fluidic precursor compared to ion diffusion. In this process, the fluidic nature of the ACP precursors allowed them to be drawn into the nano gaps and grooves of collagen fibrils via capillary infiltration [53,54]. However, whether capillary action is involved in intrafibrillar mineralization due to PILP is still debatable as this has not yet been directly observed through experiments. Exclusion of ion diffusion does not guarantee that capillary action plays a role in the intrafibrillar mineralization process. Nevertheless, capillary action has been regarded as one of the major mechanisms to date to induce intrafibrillar mineralization of collagen, although other mechanisms may have also been involved [1,24,28,55,56,57].

### 3.2. Electrostatic Attraction

The phenomenon of electrostatic attraction in collagen mineralization has been recognized as early as last century [58]. Not until a decade ago that the role of collagen in bone apatite mineralization was convincingly revealed [31], had electrostatic attraction been fully accepted as one of the major mechanisms in inducing intrafibrillar mineralization of collagen fibrils. With a combination of cryo-TEM, cryogenic electron tomography and molecular modeling, Nudelman el at. [31] found that the positive net charge close to the C-terminal end of collagen molecules promotes the infiltration of ACP into collagen fibrils. Moreover, they revealed that the clusters of charged amino acids in the gap and overlap zones formed nucleation sites for apatite crystallization converted from ACP, giving a parallel array of oriented apatite crystals to collagen fibrils. At the same time, they identified the negative net charge of PASP stabilized ACP precursors. Based on these findings, they opined that, in vivo, negatively charged NCPs might not only stabilize the amorphous phase by inhibiting apatite nucleation, but also play an active role in aiding the formation of negatively charged complexes of mineral precursors that allowed the minerals to enter the collagen through electrostatic attraction [31,59]. The predominant role of collagen in apatite nucleation, growth, structure and orientation during bone mineralization was thoroughly investigated in a subsequent study reported by Wang et al. [60]. They discovered that the collagen matrix controls the size and three-dimensional distribution of apatite, further supporting the mechanisms proposed by Nudelman et al. Thereafter, electrostatic attraction has become a popular mechanism and has been accepted in many studies [61,62,63]. In some of our work, we also believe that electrostatic attraction is one of the mechanisms in inducing intrafibrillar mineralization while polyanionic analogs of NCPs are used to stabilize the amorphous precursors of minerals, though other mechanisms such as capillary action and size exclusion theories may have also played a role [24,28,64,65]. Nevertheless, the mechanism alters while polycationic analogs are involved in the reaction [66].

In recent years, there are more and more experimental observations suggesting that the collagen intrafibrillar mineralization process cannot be simply explained by electrostatic attraction. For example, it is hard to explain why site specificity exists in different parts of turkey leg tendons as some parts are heavily mineralized while some are never mineralized [67], giving that positively charged zones are distributed periodically on the surface of the entire collagen molecules. It is also incapable of explaining why electrostatic interaction occurs only at a-band of collagen molecules while both the a-band and c-band exhibit positively charged regions [68]. It has been found in a few studies that cationic polyelectrolyte, such as polyallyamine hydrochloride (PAH), could also stabilize amorphous precursors of mineral crystals and induce intrafibrillar mineralization thereafter [66,69,70]. These cationic polymers are unlikely to bind to the positive net charge of collagen fibrils through electrostatic attraction and facilitate the infiltration of amorphous precursors. A recent work conducted by Song et al. [71] indicated that the zeta potential of collagen bound with high-molecular weight PAA (450 kDa) was –17.17 ± 1.98 eV, while that of CaP precursors was –0.88 ± 0.07 eV. Apparently, attachment of these two negatively charged species cannot be simply explained by electrostatic attractions according to the basis of Coulombic interpretations.

### 3.3. Size Exclusion

The size exclusion characteristics in collagen mineralization was observed in 2007 by Price et al. [72]. It was derived from the theory that the collagen fibrils were formed first and then water compartment within the fibrils was replaced with minerals during mineralization. It was proposed that collagen not only provided the position for mineral deposition and growth, but also acted as a gatekeeper to screen different molecules and determine whether they could diffuse into the inner spaces of collagen fibrils. A gel filtration-like column chromatography was used in the study to investigate the size exclusion characteristic of type I collagen extracted from bovine Achilles tendon. The results demonstrated that molecules smaller than 6 kDa could diffuse into all of the water compartment within the collagen fibril, whereas molecules larger than 40 kDa were excluded from this liquid. This means protein molecules smaller than 6 kDa, such as osteocalcin (OCN; 5.8 kDa) and bone Gla protein (BGP; 5.7 kDa), can interact directly with minerals and grow within collagen fibrils, while those larger than 40 kDa, such as fetuin (48 kDa) and albumin (66 kDa), cannot [32,73]. Importantly, Price et al. [73] suggested that although large molecules could not directly penetrate into collagen fibrils, some large proteins could still facilitate collagen intrafibrillar mineralization via other mechanisms. For example, osteopontin (OPN; 60–65 kDa) and fetuin, can direct intrafibrillar mineralization by inhibiting apatite nucleation and growth everywhere but within collagen fibrils [55,74,75,76]. While some other large molecules, such as bone sialoprotein (BSP; 60–80 kDa), can generate apatite crystals outside collagen fibrils, and these small crystals then diffuse into the gap zone of collagen and grow to replace water compartment inside collagen fibrils, thereby forming intrafibrillar mineralized collagen [75,77]. This theory was termed “mineralization by inhibitor exclusion”.

In a recent work, Niu et al. [70] examined this theory in PAH-directed collagen intrafibrillar mineralization where electrostatic attraction theory was not applicable as discussed in the last section. They found that although amine-containing PAH (15 kDa) could only access to 50.5% of the intrafibrillar water, it was still able to act as a nucleation inhibitor and produce excellent intrafibrillar mineralization. Interestingly, when another amine-containing small molecule, spermine (202 Da), which can access 98.64% of intrafibrillar water, was added to PAH-containing mineralization solution, the rate of collagen intrafibrillar mineralization was significantly reduced. Hence, the rate or degree of intrafibrillar mineralization induced by NCPs is more likely dependent on the nature of NCPs, such as their ability to inhibit nucleation of CaP, rather than their size or accessibility to water compartment within collagen fibrils. This was also evidenced in one of our recent works, where carboxyl-rich brush-like polymers were used to induce collagen intrafibrillar mineralization [64]. Two large-molecular, brush-like polymers with enriched carboxyl branches, carboxylated polyethylene glycol terpolymer (PEG-COOH; 42 kDa) and polyethylene glycol/polyacrylic acid copolymer (PEG-PAA; 44 kDa), were synthesized in house. Compared to linear PAA (2 kDa), which theoretically has full access to the intrafibrillar water, both brush-like polymers have demonstrated better capability to induce intrafibrillar mineralization, which might be attributed to the high availability of carboxylic groups of these polymers [61,78,79]. These results partially revealed the limitation of size exclusion theory in intrafibrillar mineralization but also endorsed the selective permeability of collagen.

### 3.4. Gibbs-Donnan Equilibrium

After verifying the selective permeability of collagen in PAH-directed mineralization, Niu et al. [70] compared the effect of polycationic and polyanionic electrolyte in directing intrafibrillar mineralization. Based on experimental observations and molecular dynamic simulations, they proposed that in polyelectrolyte-directed (both polycation- and polyanion-directed) collagen mineralization systems, Gibbs-Donnan equilibrium had to be reached between the outside (extrafibrillar) and inside (intrafibrillar) spaces of collagen fibrils via simultaneously balancing electrostatic neutrality and osmotic equilibrium. Since no difference was observed while either polycationic or polyanionic electrolyte was used to induce collagen mineralization, they concluded that electrostatic attraction was not the only mechanism for inducing intrafibrillar mineralization of collagen suggesting that additional driving forces might have controlled the movement of prenucleation clusters (classical theory) or ACP (non-classical theory) toward collagen fibrils. Osmosis describes how water moves from a low concentration to a higher concentration region across a semipermeable membrane [80]. While collagen is evidenced to have selective permeability, the osmolality of PAH in mineralization solution (ACP solution) and water was compared. It was found that the osmolality of PAH-ACP is about 20-fold as high as that of PAH in water at the same concentration. Hence, due to concentration differences of osmotically-active species and salt ions (Na^+^, Cl^–^) within and outside of collagen fibrils, it is reasonable to assume that the high oncotic pressure between the intrafibrillar and extrafibrillar spaces in the presence of a selectively permeable membrane (collagen) could provide a long-ranged interaction for ACP to infiltrate into fibrillar collagen. In one of their subsequent work, Ma et al. [47] further highlighted experimentally the importance of establishing Gibbs-Donnan equilibrium in forming intrafibrillar mineralization of collagen in a PASP and PAH dual-analog-directed mineralization system.

Overall, the establishment of Gibbs-Donnan equilibrium suggested that both short-range electrostatic forces driven by charge distribution and long-range osmosis driven by osmotic pressure between the extrafibrillar and intrafibrillar water compartments are responsible for collagen mineralization. The work provided significant insight of the effect of driving forces on ACP infiltration and for the first time disclosed the crucial role of osmotic forces in collagen mineralization [75,81,82]. It opened a new pathway in explaining collagen mineralization mechanisms. Despite of the great breakthrough, only partial in vitro models and molecular simulations were employed in these studies, so there is a need to develop direct experiments to support these theories. Moreover, none of these studies have ruled out the role of capillary infiltration in intrafibrillar mineralization, which is believed to provide rapid and long-range transportation of either prenucleation clusters or fluidic amorphous precursors [50,83,84,85]. Still, the question raised in Section 3.2 regarding the site specificity of mineralization observed in turkey tendon is not answered by the Gibbs-Donnan equilibrium theory.

### 3.5. Interfacial Energy Guided Mineralization

Recently, Kim el at. [23] proposed an intrafibrillar mineralization mechanism directed by nucleation energy barrier. By using in situ small-angle and wide-angle X-ray scattering (SAXS and WAXS) observations and classical nucleation theory, they found that PASP could increase interfacial energy between CaP nuclei and mineralization fluid. In contrast, the confined gap region within collagen fibrils could decrease the energy barrier by reducing the reactive surface area of CaP nuclei and corresponding surface energy penalty. Therefore, it was believed that the confined gap geometry guided the two-dimensional morphology and structure of apatite and changes of nucleation pathway by reducing the total energy barrier for intrafibrillar mineralization. Their work for the first time calculated the free energy change per molecule (∆G) during apatite nucleation and the interfacial energy (α) between CaP nuclei and mineralization solution, for intrafibrillar and extrafibrillar mineralization, respectively, at different nucleation sites. Another work regarding interfacial energy guided collagen mineralization was reported by Shao et al. [86] in a citrate-aided intrafibrillar mineralization system. It was found that the adsorbed citrate molecules on collagen fibrils significantly reduced the interfacial energy between the collagen matrix and the ACP precursor via interface wetting effect, and therefore facilitating intrafibrillar mineralization of apatite at an early stage of biomineralization. Similarly, polydopamine (PDA) was utilized in their subsequent work by Qu et al. [87] to promote dentin remineralization with decreased heterogeneous nucleation energy barrier by reducing interfacial energy of collagen/ACP.

Overall, these studies further advanced our understanding on the interactions between collagen fibrils and minerals during intrafibrillar mineralization, and quantitatively emphasized the importance of reducing interfacial energy of collagen/ACP to promote heterogeneous nucleation, which could potentially enhance mineral formation within collagen fibrils. However, the proposed mechanism still has some limitations. For instance, the molecular weight of PASP that Kim et al. used was about 5 kDa, which is supposed to be completely permeable to intrafibrillar water compartment [72]. Thus, the interfacial energies at both the extrafibrillar and intrafibrillar spaces would be affected by PASP, but it was assumed that the addition of PASP only kinetically inhibited the formation of extrafibrillar mineralization. Moreover, the calculation of heterogenous nucleation barrier relied largely on the classical nucleation theory, which is still debatable from the point of view of non-classical theory [88,89].

## 4. Biomimetic Intrafibrillar Mineralization Strategies for Hard Tissue Repair

The high strength of mussel shells and seashells reflects how nature applies nanotechnology to strengthen load-bearing hard tissues, and biomineralization of collagen is an excellent example [27,90]. Conversely, self-assembly of collagen-based materials through biomimetic mineralization is a nanotechnology inspired by nature. It provides an effective approach to develop advanced biomaterials in tissue engineering with abilities to not only control the functions of osteoblasts and bone marrow mesenchymal cells (BMSCs), but also guide and promote new bone regeneration in defined shapes [91]. Compared to pure collagen and extrafibrillar mineralized collagen, collagen-based tissue engineering scaffolds composed of intrafibrillar mineralized fibrils demonstrated solid advantages, such as enhanced mechanical property, increased in vitro osteogenesis and in vivo bone healing [92]. Intrafibrillar mineralized collagen was also thought to promote bone regeneration via activation of the Wnt signaling pathway [93]. The acceleration of dentin remineralization through various biomimetic strategies have also been reported recently to be an effective approach for tooth defect repair [94,95,96,97]. In this section, typical strategies for biomimetic synthesis of intrafibrillar mineralized collagen-based materials are included.

### 4.1. NCPs

Besides HA and collagen, there are various kinds of NCPs and extracellular molecules existing in natural bone. Hence, it is reasonable to believe that biomineralization is regulated by these components. As discussed above, collagen has been suggested to be responsible for the deposition of apatite minerals even at the absence of other factors, such as NCPs [23]. However, based on most published studies, NCPs are believed to mediate vertebrate mineralization associated with collagen by influencing the formation and maintenance of the mineralized matrix [98]. OCN is the most abundant NCP in bone influencing matrix mineralization and global metabolism that exclusively produced by osteoblasts [99]. Simon el at. [100] first evidenced the importance of this protein using high resolution TEM and Fourier analysis during collagen triple helix fibril mineralization. It was found that OCN was directly bonded with octacalcium phosphate and was often seen spreading between and over collagen fibrils like pearl necklace strings or as single spherical particles. This study revealed experimentally that OCN is attached to the collagen structure and interacts with the Ca-sites on the (100) plane dominated HA platelets. Wang et al. [75] found that in the absence of OCN, rapid and random extrafibrillar mineralization of flakey CaP particles was observed mainly on the surfaces of collagen fibrils through TEM. In contrast, hydrated, spherical nanoclusters of CaP were observed on the surface of collagen fibrils when OCN was added. These nanoclusters then infiltrated into the fibrils, forming intrafibrillarly mineralized collagen with HA nanocrystals aligned within the collagen fibrils. Recently, OCN has been found to be key to the alignment of apatite crystallites during mineralization [101]. OPN is another important NCP that mediates various biological events involving the immune and the vascular systems [102]. It was used to induce collagen intrafibrillar mineralization by serving as a process-directing agent [62]. In this study, OPN was also found to promote the interaction of mouse marrow-derived osteoclasts with re-mineralized bone, contributing to both biomineralization and bone remodeling. Such prepared intrafibrillarly mineralized materials therefore have demonstrated great potential in bone tissue engineering. Meanwhile, França et al. [55] engineered intrafibrillarly mineralized scaffolds through an OPN-guided PILP process to reproduce the nanostructure of bone. The obtained scaffolds had a similar mineral/matrix ration to native bone and its compressive modulus was increased up to 15-fold compared to pure collagen without mineralization. Although other natural NCPs, such as fetuin-A, BSP, detin matrix protein 1 (DMP1) and dentin sialophosphoprotein (DSPP), were revealed to mediate intrafibrillar mineralization [88,103,104,105], limited studies have been conducted to directly use these proteins to synthesize intrafibrillarly mineralized materials for orthopedic and dental applications due to their low availability and high cost.

### 4.2. Polymer Analogs

To overcome the limitations of natural NCPs, synthetic polymer analogs of NCPs are usually used to replicate their functions and induce collagen intrafibrillar mineralization. The use of polymers in collagen mineralization is generally considered to be achieved through a PILP process while the intrafibrillar mineralization process can be explained in one or more different mechanisms as discussed in the above section. Table 1 summarizes some recent examples of commonly used polyanionic and polycationic polymers in directing collagen intrafibrillar mineralization and their major contributions in biomineralization, bone regeneration and tooth repair.

### 4.3. Small Molecules and Fluid Shear Stress

Some small molecules, such as citrate and fluoride, have been discovered to affect intrafibrillar mineralization. Citrate is widely found in biological hard tissues such as bone and is believed to be an osteopromotive factor in mediating citrate metabolism and its downstream effects on the osteogenic differentiation of BMSCs [118]. It has also been reported that the citrate content in bone is reduced in osteoporotic and aged animals [119]. As summarized in Section 3.5, the influence of citrate on biomineralization has been well studied [86]. Citrate molecules could significantly reduce the interfacial energy between the collagen matrix and the ACP precursors, thereby facilitating intrafibrillar mineralization. Experimental results indicated that only collagen fibrils containing similar level of bound citrate to natural bone (~8.2%) could reach full mineralization comparable to bone [86]. Recently, citrate has been incorporated into collagen fibrils during their fibrillogenesis to promote intrafibrillar mineralization [63]. It was found that citrate-functionalized collagen fibrils underwent extensive intrafibrillar mineralization within 12 h in m-SBF. Another small molecule, fluoride, also showed to influence the mineralization of collagen. Different concentrations of NaF were added to the mineralization solution. As the fluoride concentration increased, the crystals became larger and more rod-like, with an increasing tendency to form fluorapatite on the fibril surfaces rather than the interior, therefore forming extra- fibrillar mineralization dominated collagen fibrils [56]. However, as fluorapatite has a lower solubility and higher hardness than HA, and thus is less likely to be dissolved in aqueous or mild acidic environment, it has been widely used in enamel and dentin remineralization to reduce the chance of cavity formation [120,121]. Although enamel does not contain collagen like bone and dentin, the addition of fluoride in collagen mineralization overall showed bright future for dental repair.

Additionally, Niu and Du et al. [122,123] shared their recent findings on collagen intrafibrillar mineralization induced by fluid shear stress (FSS). FSS is considered as a predominant stimulus to bone cells and matrix, but its role in biomineralization has not been well investigated. They discovered that the application of a small amount of FSS (less than 2 Pa, especially within 1.5 Pa) has a positive influence on mineralization by enhancing collagen self-assembly and accelerating ACP formation and transition. The capability of FSS in inducing intrafibrillar mineralization is comparable to that of commonly used NCPs analogs, PAA. Moreover, under the action of templating analogs (such as TPP), periodic FSS can also promote the formation of highly oriented hierarchical intrafibrillarly mineralized collagen, giving D-banding patterns. Their work opened a new avenue in investigation of biomineralization mechanisms by introducing vascular flow in in vitro models. It also provided a novel biomimetic strategy to induce intrafibrillar mineralization without the addition of NCPs and biomolecules.

## 5. Conclusions and Perspectives

Natural bone and dentin are organic-inorganic composites mainly consisting of collagen and HA that are arranged in multilevel hierarchical structures from atomic- to macro-scale. Their excellent mechanical property and favorable biocompatibility are largely determined by the biomineralization of collagen fibrils. To have a better understanding on collagen biomineralization and provide insight on the design of mineralized collagen-based materials, this review summarized current collagen mineralization mechanisms and recent biomimetic strategies for bone regeneration and tooth repair. Overall, these proposed mechanisms are either derived from the classical ion-cluster-based or non-classical amorphous precursor-based theories. A complementary proposal to both the classical and non-classical theories could be a future trend in establishing new mechanisms or perfecting existing ones. In addition, in vitro models involving more complicated conditions, such as fluid flow and cell loading, that better mimic the in vivo environment could provide clearer and more realistic cognition on biomineralization. The improvement of bioinspired collagen-based materials could start from the development of a simple, reliable, reproducible, and costless collagen extraction process. Despite extensive efforts, to date, only simplified biomimetic structures have been achieved. Therefore, biomaterials better mimicking the complex hierarchical natural hard tissues may be achieved using newly developed techniques such as 3D printing in combination with biomimetic strategies. Appropriate incorporation of essential elements, drugs, or biomolecules into well-designed 3D collagen-based structure during biomimetic mineralization could also be a direction to promote their performance for bone regeneration and tooth repair.

## Figures and Tables

**Table 1:** Recent examples of synthetic polymer analogs used to mediate biomimetic intrafibrillar mineralization of collagen.

Polymers	Major Findings	Ref.
PAA	Systematically investigated the effect of molecular weight and concentration of PAA on the rate and pattern of collagen intrafibrillar mineralization. Decreasing PAA molecular weight (2 kDa) and increasing PAA concentration (50 mg/L) resulted in increased stability of ACP solution and therefore enhanced intrafibrillar mineralization.	[106]
PAA or carboxyl-rich brush-like polymers; TPP	Two brush-like polymers derived from PEG were synthesized and modified with enriched carboxylic groups. Sodium tripolyphosphate (TPP) was used as a templating analog. The mineralization degree of collagen fibrils induced by brush-like polymers was higher than those induced by linear PAA. Therefore, the formation of intrafibrillarly mineralized apatite might be partially determined by the availability of carboxylic groups in the nucleation inhibitors.	[64]
PAA; TPP	Intrafibrillarly mineralized collagen-based scaffolds were engineered into both cellular and lamellar structures. Fe and Mn were incorporated separately or jointly into the lamellar scaffold. Lamellar scaffolds were much better in supporting in vitro osteogenic differentiation and in vivo bone regeneration than cellular scaffolds. These promotion effects were further enhanced by the addition of both Fe and Mn ions.	[65]
PASP	PASP chain length contributed to the effectiveness of mediating intrafibrillar mineralization. The process appeared to be associated with the inhibition of apatite crystallization by PASP through slowing the growth of ACP stabilizing this phase.	[81]
CMC	Collagen scaffolds with both intrafibrillar and extrafibrillar mineralization were obtained using carboxymethyl chitosan (CMC) to stabilize ACP. Such prepared scaffolds exhibited increased modulus, in vitro cell proliferation and differentiation, and in vivo new bone regeneration compared to unmineralized collagen scaffolds as well as those fabricated using traditional modified-simulated body fluid (m-SBF) solution without CMC.	[107]
CMC	Sr- and Ag-doped intrafibrillarly mineralized collagen scaffolds were developed. Ag-doped scaffolds showed enhanced antibacterial effect on S. aureus while Sr-doped scaffolds illustrated enhanced new bone regeneration.	[108]
Pchi	Phosphorylated chitosan (Pchi) was used to promote collagen intrafibrillar mineralization through a biomimetic approach. It was found that Pchi significantly shortened the self-assembly process by accelerating the rate of crystallization due to the excellent ion chelating properties of chitosan derivatives and their ability to induce high-degree conglutination.	[109]
PAMAM	Carboxyl-terminated hyperbranched polyamidoamine dendrimer (PAMAM) was used to induce biomimetic remineralization on dentine organic matrix. Such prepared material showed great potential to be used as a new therapeutic material for the treatment of dentin hypersensitivity.	[110,111]
PDA	Dentine was successfully repaired using polydopamine (PDA), which promoted intrafibrillar mineralization by decreasing the interfacial energy between collagen matrix and ACP. The re-mineralized dentin exhibited comparable mechanical properties to natural dentin.	[87]
CS	The immobilized chondroitin sulfate (CS) on collagen fibrils accelerated CaP nucleation and significantly promoted collagen intrafibrillar mineralization by providing specific sites for CaP nucleation within the collagen fibrils. Remarkably accelerated remineralization of CS immobilized demineralized dentin was achieved.	[112]
Alginate	As an anionic polyelectrolyte with Ca-capturing capacity, alginate was used to successfully mediate intrafibrillar mineralization of collagen. The alginate-assisted mineralization of collagen resulted in an exquisite three-dimensional (3D) mineralized architecture with enhanced mechanical properties as well as excellent proliferation, adhesion, and differentiation of rat BMSCs.	[113]
PAH; TPP	Intrafibrillarly silicified collagen scaffolds were prepared in the presence of PAH as a directing agent. The silicified scaffolds supported in vitro cell proliferation, in situ bone regeneration and angiogenesis via monocyte immunomodulation.	[66,114]
Peptide	Various kinds of mineral-promoting peptides with different amino acid sequences were designed to mimic the functions of NCPs to enhance intrafibrillar mineralization of collagen. The obtained materials displayed: (1) stronger influence on biomineralization than traditional used PAA; (2) excellent ability for rapid remineralization of dentin; or (3) apparent improvement in restoring incipient enamel decay and mineralization defects localized in peripheral dentin.	[115,116,117]

## Abbreviations

HA	Hydroxyapatite
Pro	Proline
Hyp	Hydroxyproline
Gly	Glycyl
ECM	Extracellular matrix
Col-HA	Collagen-hydroxyapatite
CaP	Calcium phosphate
NCPs	Non-collagenous proteins
ACP	Amorphous calcium phosphate
PILP	Polymer induced liquid precursor
ACC	Amorphous calcium carbonate
PASP	Polyaspartic acid
cyro-TEM	Cryogenic transmission electron microscopy
AFM	Atomic force microscopy
NMR	Nuclear magnetic resonance
PAA	Polyacrylic acid
SEM	Scanning electron microscopy
TEM	Transmission electron microscopy
XRD	X-ray diffraction
PAH	Polyallyamine hydrochloride
OCN	Osteocalcin
BGP	Bone Gla protein
OPN	Osteopontin
BSP	Bone sialoprotein
PEG-COOH	Carboxylated polyethylene glycol terpolymer
PEG-PAA	Polyethylene glycol/polyacrylic acid copolymer
SAXS	Small-angle X-ray scattering
WAXS	Wide-angle X-ray scattering
PDA	Polydopamine
TPP	Sodium tripolyphosphate
CMC	Carboxymethyl chitosan
m-SBF	Modified-simulated body fluid
Pchi	Phosphorylated chitosan
PAMAM	Polyamidoamine dendrimer
CS	Chondroitin sulfate
3D	Three-dimensional
FSS	Fluid shear stress
